# Recombinase Polymerase Amplification Assay with and without Nuclease-Dependent-Labeled Oligonucleotide Probe

**DOI:** 10.3390/ijms222111885

**Published:** 2021-11-02

**Authors:** Aleksandr V. Ivanov, Irina V. Safenkova, Anatoly V. Zherdev, Boris B. Dzantiev

**Affiliations:** A.N. Bach Institute of Biochemistry, Research Centre of Biotechnology of the Russian Academy of Sciences, Leninsky Prospect 33, 119071 Moscow, Russia; a.ivanov@fbras.ru (A.V.I.); safenkova@inbi.ras.ru (I.V.S.); zherdev@inbi.ras.ru (A.V.Z.)

**Keywords:** RNA virus, RPA probe with tetrohydrofuran, lateral flow test, nfoRPA

## Abstract

The combination of recombinase polymerase amplification (RPA) and lateral flow test (LFT) is a strong diagnostic tool for rapid pathogen detection in resource-limited conditions. Here, we compared two methods generating labeled RPA amplicons following their detection by LFT: (1) the basic one with primers modified with different tags at the terminals and (2) the nuclease-dependent one with the primers and labeled oligonucleotide probe for nuclease digestion that was recommended for the high specificity of the assay. Using both methods, we developed an RPA-LFT assay for the detection of worldwide distributed phytopathogen—alfalfa mosaic virus (AMV). A forward primer modified with fluorescein and a reverse primer with biotin and fluorescein-labeled oligonucleotide probe were designed and verified by RPA. Both labeling approaches and their related assays were characterized using the in vitro-transcribed mRNA of AMV and reverse transcription reaction. The results demonstrated that the RPA-LFT assay based on primers-labeling detected 10^3^ copies of RNA in reaction during 30 min and had a half-maximal binding concentration 22 times lower than probe-dependent RPA-LFT. The developed RPA-LFT was successfully applied for the detection of AMV-infected plants. The results can be the main reason for choosing simple labeling with primers for RPA-LFT for the detection of other pathogens.

## 1. Introduction

Recombinase polymerase amplification (RPA) is an isothermal approach that is actively applied for the field and low-equipment detection of DNA or RNA targets [1,2]. RPA is based on recombinase-dependent hybridization of primers with a target DNA, followed by strand-displacing DNA polymerization by BsuI polymerase. This generates plenty of double-stranded (ds) DNA amplicons at 37–42 °C for 15–20 min [3]. The addition of reverse transcription (RT) reaction to RPA allows the use of RNA molecules as target molecules. The amplicons can be detected in different ways: visualization by gel electrophoresis, by real-time detection using fluorescent dye or probes, and by specific and affine recognition of tag-labeled amplicons in lateral flow test (LFT) [1,3]. The LFT is the most popular tool for sensitive and rapid detection of amplicons that is effective in resource-limited conditions [4]. To realize RPA-LFT, two different specific tags should be included in the amplicons during the RPA process. The tags can be low molecular, such as fluorescein (FAM), biotin, digoxygenin, etc., or a single-stranded (ss) DNA tail. The tags are affinely captured by receptor molecules at LFT that are located at specific zones and interact with tag-labeled amplicons that pass through the test strip [5,6,7].

**Figure 1:**
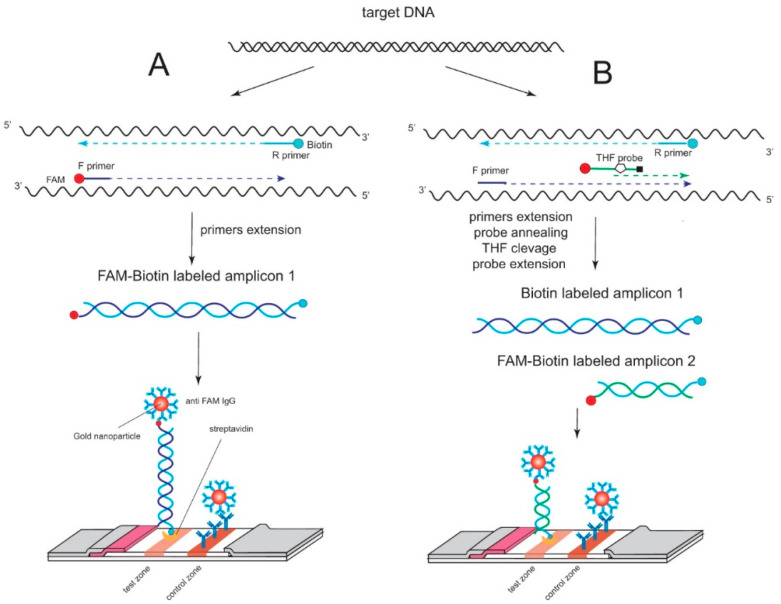
Scheme of RPA-LFT with the generation of labeled amplicons and their detection, based on (**A**) both labeled primers, (**B**) labeled THF probe, and labeled reverse primer.

The easiest way to obtain the labeled amplicon is by using forward and reverse RPA primers labeled at the 5′ ends with different tags (the scheme is presented in Figure 1A). This approach is actively applied for the RPA-LFT detection of pathogens and species identification [8,9,10,11]. However, the probability of the cross-dimerization of the labeled RPA primers is high due to the typical RPA primers being rather long (28–35 nt), and there are no stages with the temperature denaturation of DNA. Cross-dimers can lead to false positive signals for several reasons. Thus, labeled cross-dimers can be bound in the test zones or can be used as an RPA template for the synthesis of the by-product-labeled amplicon. To solve this problem, Piepenburg et al. [3] suggested using an additional oligonucleotide probe with a tag to reduce the nonspecific product. This approach is widely used in RPA-LFT assays [12]. The oligonucleotide probe is annealed within the region limited by primers and usually does not overlap with primers. The probe consists of tag (e.g., FAM) at 5′ end; 30–35 nt oligonucleotide, tetrahydrofuran (THF) residue, which replaces a nucleotide; short (approximately 15 nt) oligonucleotide; and 3′-blocking modification (e.g., phosphate or PEG) [3] (see Figure 1B). This approach supposes that RPA forward primer is unmodified, and the reverse has a tag (e.g., biotin). The probe specifically binds to the synthetized amplicon after the THF site is recognized and destructed by Nfo endonuclease IV, and 30–35 nt oligonucleotide part acts as a primer for BsuI polymerase, replacing the short part of the probe with blocking modification [13]. As a result, two populations of amplicons were synthesized, with one tag from a reverse primer that is not detected by LFT and a secondary one with two tags detected by LFT (see scheme in Figure 1B). The probe is unable to form a stable complex with a nonspecific amplicon. This significantly decreases the formation of labeled by-product amplicons [14]. Some research proposed a solution for decreasing the false-positive signal of RPA-LFT by the optimization of the probe design [15], using blocking antiprimers [16] and the proper combination of probe–primer sets [17,18] to avoid their cross-dimerization. However, there is no research about the direct comparison of RPA-LFT based on the generation of labeled amplicons through forward and reverse primers and through the oligonucleotide probe and reverse primer. In this study, a comparison of both ways with the same target and considering specificity and sensitivity of the RPA-LFT was performed for the first time.

Alfalfa mosaic virus (AMV), a positive ssRNA virus that belongs to the *Bromoviridae* family, is a worldwide distributed phytopathogen [19,20] and was chosen as a target for RT-RPA-LFTs with both labeling approaches. AMV has caused outbreaks around the world and infected more than 150 plant species, damaging the harvest of different varieties, such as soybean [21,22], lucerne [23,24], potato [25,26], chayote [27], etc. The virus contains three genomic RNAs that encode four proteins. The most abundant structural protein, coat protein (CP), is coded by RNA3 and proceeds from the subgenomic RNA4 [28,29]. RNA4 is most present among the AMV RNAs in host plant cells during the infection [30,31]. There are several assays for AMV detection, including immunoassay [32,33], RT-PCR [34,35], and RT-LAMP [36]. Despite the advantages of RPA, no RPA systems (e.g., RPA-LFT) were developed for the detection of AMV RPA.

Hence, the main task of this research was the development of RT-RPA-LFTs of AMV RNA based on two different methods of labeled amplicons’ generation, comparison of their specificity and sensitivity, and a verification test system with improved analytical characteristics by using infected and healthy plant material.

## 2. Results and Discussion

### 2.1. Primers and Probe Selections for RT-RPA-LFT

First, primers for RPA were designed. Previous researchers proposed several primers pairs for PCR (see Supplementary Materials (SM), Table S1). Most of them recognized RNA3, particularly gene of AMV coat protein. RNA3 and subgenomic-proceeded RNA4 are most presented in the host cell, so they are preferred targets for detection. Moreover, some previously proposed primers were predicted to form self- or cross-dimers (SM, Table S1). From the described predecessors, primer pairs were chosen that are nondimerizing and produce amplicons shorter than 400 bp, which is demanded by RPA. The pair was named AMV F1/AMV R1 (sequences are presented in Table 1 and SM, Table S1). These primers have 21–23 nt lengths; however, the use of primers shorter than 25 nt in RPA is possible but not recommended [12]. Therefore, RPA primers AMV F3/AMV R3 (Table 1) were designed by extension of AMV F1/AMV R1 primers to make them a more appropriate length for RPA. Such primer extension can improve RPA sensitivity [10,37]. Additionally, a new pair for the AMV CP gene, AMV F4/AMV R4, was designed (Table 1). Recognition sites of the primers and the length of possible amplicons generated by these primers are presented in SM, Section S1. For the following primers, combinations that produce amplicons shorter than 400 bp were selected.

Verification of the primers in nine different combinations (F1-R1, F1-R3, F1-R4, F3-R1, F3-R3, F3-R4, F4-R1, F4-R3, F4-R4) were performed using AMV RNA3Δ (SM, Section S2) by RT-qPCR (SM, Section S3, Figure S2A,B). Due to the RT-qPCR curves being very similar for all primer pairs, RT-RPA-LFTs for all designed pairs were also carried out.

**Figure 2:**
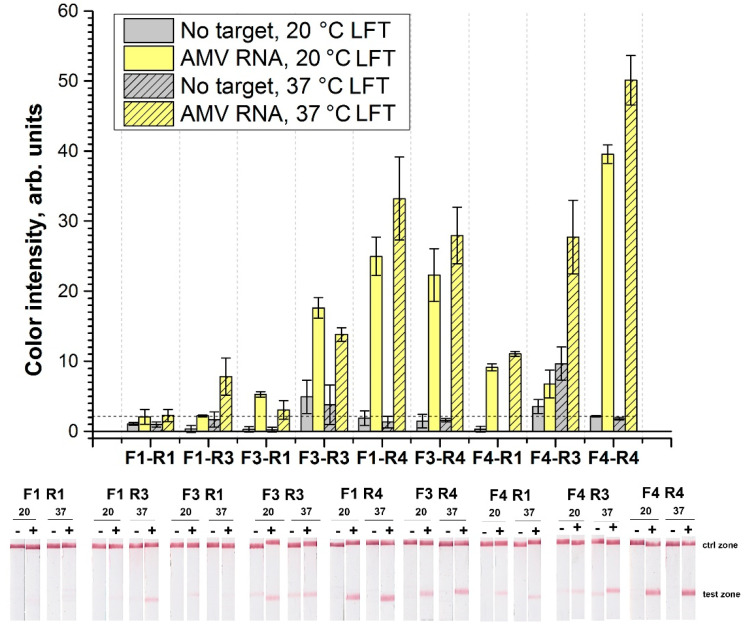
Signal in test zone of LFT strip after RPA of positive (10^7^ copies of target) and negative (TE buffer) sample controls. LFT was performed at different temperatures. The dashed line represents a signal visible to the naked eye (2 a.u.).

For RT-RPA-LFT, FAM-labeled forward primers and biotin-labeled reverse primers (see Table 1) were used in the same nine combinations. As a result, FAM- and biotin-labeled amplicons were formed during RPA and detected by LFT strip containing streptavidin immobilized at the test zone and conjugate of gold nanoparticles (GNPs) with antibodies specific to FAM. The lengths of the amplicons differed from 150 to 367 bp (SM, Section S1, Table S2) based on the selected pair. The selected pair was compared when no target RNA was added to buffer (negative sample), or 10^7^ copies AMV RNA3 were added (positive sample; Figure 2). Pair F1-R1 demonstrated negligible signals for negative and positive samples. Pair combinations F3-R1 and F4-R1 demonstrated no nonspecific signal in the RPA-LFT of negative samples. However, signals of positive samples were moderate (<10 a.u.) and within the use of these primers pairs. The other primer combinations showed pronounced signals with positive samples and weak visible signals with negative samples (See Figure 2). To improve the results, LFT was performed at 37 °C, as applied in previous research concerning RT-RPA-LFT of viruses [9]. Increasing LFT temperature enhanced the signals of positive samples with all pairs besides F1-R1 and F3-R1. However, the conditions were not eliminated, and nonspecific signals of negative controls were obtained for all pairs. The F4-R4 primers’ combination was chosen because the positive control had the highest signal, and the nonspecific signal was conterminal to the visually detectable (2 a.u.). RT-PCR based on the F4-R4 pair detected 10^4^ copies of AMV RNA3 according to agarose gel electrophoresis (SM, Section S3, Figure S3) that corresponded to RT-PCR based on the proposed earlier F1-R1 pair.

For the selected F4-R4 pair, we designed a specific probe that was annealed to DNA between the annealing sites of the primers (SM, Section S1). The probe consisted of the FAM-tag at 5′ end, 30 nt part, THF residue, 14 nt part, and 3′ blocking phosphate (Pi; see Table 1). Therefore, the probe composition and location fully complied with the recommendations [3] and can be generated together with the R4 primer FAM- and biotin-labeled ds amplicon with a length of 99 bp.

### 2.2. Characterization of RT-RPA-LFTs of AMV RNA3

**Figure 3:**
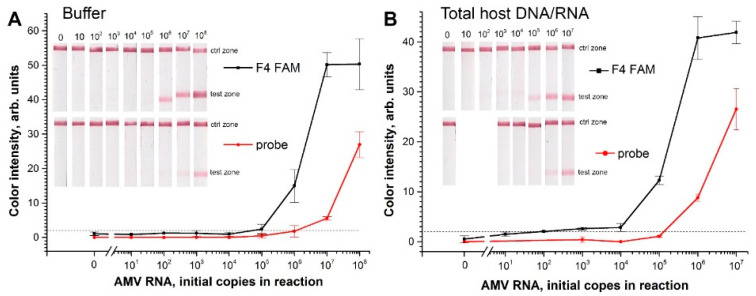
Dependences of RT-RPA-LFT signal from initial amount of synthetic AMV RNA3 performed by different ways of amplicon labeling: (**A**) the target RNA diluted in TE buffer; (**B**) the target RNA in potato total DNA. The black dashed line marks visibility to the eye of the signal.

RT-RPA-LFTs with F4-R4 primers were performed based on two different methods of labeled amplicons. The first method was based on the generation of labeled RPA amplicons with a length of 150 bp through FAM-labeled F4 primer and biotin-labeled R4 primer. The length of 150 bp was found to be optimal for LFT of FAM- and biotin-labeled dsDNA amplicon [38]. The second method was based on the generation of labeled RPA amplicons with a length of 99 bp through the FAM-labeled probe and biotin-labeled R4 primer. The F4 primer was used for amplification without the FAM tag. For both methods, the assay was carried out with different amounts of AMV RNA3Δ (from 10^8^ copies in reaction to 10 copies). RT-RPA-LFT with labeled primers detected AMV RNA3 diluted in TE buffer from 10^5^ copies in reaction (Figure 3A). The detection limit of RT-RPA-LFT based on the labeled probe was higher and equal to 10^6^ copies of RNA per reaction. The half-maximal-binding concentrations of RNA for assay based on primers labeling was 50 times lower than for assay based on probe labeling (sigmoidal fits and their parameters are given in SM, Figure S4, Table S3). There were no visually detectable nonspecific signals in either assay. In the case of the instrumental detection of RT-RPA-LFT with labeled primers, a very insignificant background was observed (see Figure 3A).

The same experiments with the target AMV RNA3Δ diluted in total DNA extracted from potato leaves were performed (Figure 3B). These experiments model the extraction of AMV RNA from infected plants. The addition of the unspecific DNA caused significant enhancement of RT-RPA-LFT for both labeling types (Figure 3). It shifted the calibration curves toward lower concentrations. The half-maximal-binding concentrations decreased 10 and 23 times for assay based on primers- and probe labeling, respectively (see SM, Section S4). The difference between the half-maximal binding of primers labeling from probe labeling in the presence of unspecific DNA was 22 times. In the case of RT-RPA-LFT based on primer labeling, the gradual increment of the signal at a low concentration of initial RNA in the range of 10–10^4^ appeared. Moreover, the addition of total DNA completely removed false-positive signal (from 1.1 ± 0.5 a.u. to 0.4 ± 0.5 a.u.) of null samples while labeled F4-R4 primers were used. These effects increased RT-RPA-LFT sensitivity to 10^3^ of initial copies of AMV RNA3Δ. The supplementation of the target RNA by total plant DNA did not have an impact on unspecific signals for RT-RPA-LFT with the THF probe (Figure 3). 

**Figure 4:**
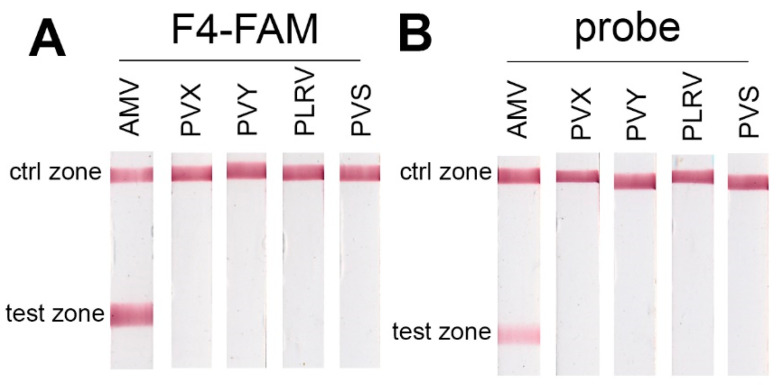
Test of specificity RT-RPA-LFT with AMV, PVX, PVY, PLRV, and PVS: (**A**) when labeled primers were used; (**B**) when THF probe was used.

Specificity of the RT-RPA-LFTs based on the primers and the probe in presence of other widespread potato viruses (PVX, PVY, PVS, and PLRV) was tested using 10^8^ copies of purified viral RNAs spiked with total potato DNA/RNA. The same amount of spiked AMV RNA3 was used as a positive control. Both methods of RT-RPA-LFT demonstrated specific signals with AMV RNA3 and no signal in the presence of other tested viruses (Figure 4).

The developed RT-RPA-LFT assay can be considered rapid. The time of RT-RPA was 20 min, followed by 10 min of LFT, in total.

These experiments clearly demonstrated that the sensitivity of the simpler method of amplicon labeling using modified primers can exceed the sensitivity of the approach with a THF probe up to three orders, as in our case. Certainly, amplification with a probe is a derivative process concerning primer-based amplification. Thus, the sensitivity of Nfo-RPA is limited by the sensitivity of basic RPA with primers only. Concurrently, there was no difference in specificity between the labeling ways (see Figure 4). The specificity and sensitivity of RT-RPA-LFT based on labeled primers increased when AMV RNA3 was added in the presence of abundance nontarget DNA concentration (approx. 200 ng/µL or 40 ng/µL in final RPA mix). RPA is known to tolerate unspecified DNA in samples [39,40]. However, there have been no data declaring the stimulating effect of total unspecified DNA on RPA or RPA-LFT as of yet. There was evidence about inhibition of RPA by the presence of a high concentration of total unspecified DNA in the RPA test with the exoRPA kit [40] and the nfoRPA kit, followed by LFT [41]. The concentration of total DNA/RNA in our experiments was not as high as one that inhibits RPA. Additionally, we used plant DNA/RNA, but human DNA could have another effect. It is feasible that DNA could weakly interact with the primers that sequester them and reduce their cross-dimerization. Another explanation could be the blocking effect of charged nucleic acid polymer to the nitrocellulose membrane in the test zone.

RT-RPA-LFT of the spiked samples had a higher sensitivity (10^3^ copies per reaction or 20 copies/µL) than RT-qPCR performed with the same primers or previously developed primers (10^4^ copies per reaction or 1000 copies/µL; see SM, Figures S2B and S3). None of the previous research provided sensitivity data for the proposed PCR tests. Hence, a comparison of analytic characteristics of the developed test system is unfeasible. Based on the experience of previous works on the detection of other RNA plant viruses [9,11,42,43], we considered the developed test as appropriate for the detection of real AMV in infected plants.

### 2.3. Verification of the RT-RPA-LFT by Testing AMV Infected Plants

**Figure 5:**
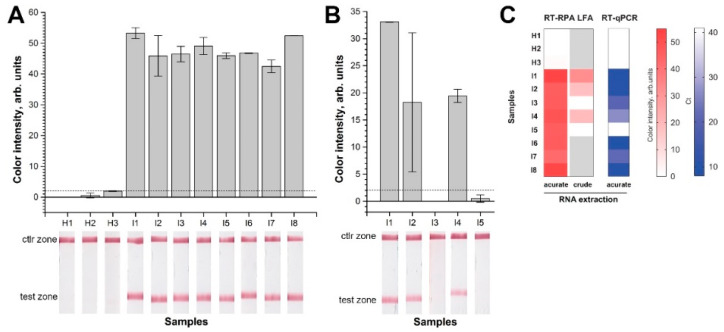
Verification of RT-RPA-LFT test for AMV detection. H—healthy samples, I—infected samples: (**A**) extraction of total DNA/RNA by kit; (**B**) rapid crude extraction of DNA/RNA by grinding; (**C**) comparison of RT-RPA-LFT and RT-qPCR with samples from healthy and infected plants. The results for each sample are presented as distinct squares in the heat maps.

To verify the developed RT-RPA-LFT system, total nucleic acid was extracted from the leaves of healthy potato plants (H1-H3), and from the leaves of potato (I1, I2, I4, I6, I7, I8) and tobacco (I3, I5) that were artificially infected with AMV. The RT-RPA-LFT was performed with the samples using FAM-labeled F4 and biotin-labeled R4 primers because this method tends to be more sensitive and specific in previous experiments. The assay detected none or minimal nonsignificant signals when healthy samples were used (Figure 5A). All samples of AMV-infected plants produced pronounced positive signals in RT-RPA-LFT (Figure 5A). Moreover, the RPA-LFT assay was checked for AMV detection in some infected samples after fast crude extraction. RT-RPA-LFT was able to detect the AMV even in samples after this crude extraction: 1 min maceration in plastic bags with plastic mesh, and with the addition of a TE buffer (Figure 5B). However, only potato samples (I1, I2, I4) were detectable, whereas both tobacco samples (I3, I5) showed no signal. Additionally, the signal of some positive samples had high dispersion. Feasibly, tobacco contains some metabolites that affected RT-RPA or made the structure of the tobacco leaf more resistant to grinding and prevented the release of viral RNA. The results of the developed RT-RPA-LFT were correlated with the results of RT-qPCR (Figure 5C and SM, Section S5, Figure S5).

Therefore, the developed system can be used for the sensitive, specific, and rapid diagnostics of AMV infection, particularly in potato plants. As the application of the oligonucleotide probe for nuclease digestion is dispensable, it makes the RT-RPA-LFT more accessible for field diagnostics because it avoids using a nfoRPA kit or a complex synthesis of the probe. Therefore, the developed RPA-LFT is a disposable device and allows one analysis to be performed under any conditions, including in-field diagnostics.

## 3. Materials and Methods

### 3.1. Reagents

Kits for Nfo-RPA were manufactured by TwisDx (Maidenhead, UK). Unlabeled primers were synthesized by Evrogen. Biotin- and FAM-labeled primers were synthesized by Syntol (Moscow, Russia). Kits of total DNA/RNA extraction from plants and an RNAse inhibitor (RNAsin) were purchased from Syntol. A FAM-labeled THF-containing oligonucleotide probe was synthesized by BioResearch (Risskov, Denmark). The mix for PCR contained SYBR Green I, dNTP, Tersus polymerase, and Moloney murine leukemia virus (MMLV) revertase, and the kits for DNA extraction from gels were purchased from Evrogen (Moscow, Russia). T7 RNA polymerase, DNAse I, RNA cleanup kit, and NTPs were purchased from Neb (Ipswich, MA, USA). Mouse monoclonal IgG (clone 2A3c) specific to fluorescein (anti-FAM) was produced by Bialexa (Moscow, Russia). Recombinant streptavidin and goat anti-mouse IgG were produced by Imtek (Moscow, Russia). Ethylenediaminetetraacetic acid (EDTA), HAuCl_4_, bovine serum albumin (BSA), and dithiothreitol (DTT) were purchased from Sigma-Aldrich (St. Louis, MI, USA). Salts, buffers, organic solvents, and other compounds were analytical grade.

For LFT-producing nitrocellulose membrane CNPC12, PT R5 fiberglass membrane, sample pad membrane GFB-R4, and absorbent pad AP045 were purchased from Advanced Microdevices (Ambala Cantt, India).

### 3.2. In Vitro Transcription of AMV RNA3

A sequence containing the AMV RNA3 CP (GenBank accession K02703) gene flanked with the T7 promoter and the T7 terminator was synthesized and cloned in a pCORE 006 vector by Cloning Facility (Moscow, Russia). A sequence of the CP gene without the flanks is given in SM, Section S1. The DNA template for in vitro transcription was synthesized by PCR. The PCR was performed using 200 nM pCORE F (5′-CTCGACGCTGCCGAGATTGC-3′) and AMV RNA3 R (5′- GCATCCCTTAGGGGCATTCATGC-3′) primers and 2 µM dNTP, 5 u. of Tersus polymerase, and corresponding Tersus buffer. PCR cycles comprise the denaturation cycle at 95 °C for 20 s, primers annealing at 60 °C for 20 s, and elongation at 72 °C for 60 s. PCR was carried out within 40 cycles. The target DNA amplicon of the RNA3 gene was purified with 1% agarose gel electrophoresis, followed by the DNA extraction by the DNA gel extraction kit. The DNA fragment containing a T7 promoter, and an RNA3 gene was used as a template for “runoff” with an in vitro transcription. The DNA fragment was added to up to 165 ng/µL (290 nM) of reaction mix containing 166 mM HEPES-KOH pH 7.5, 32 mM MgCl_2_, 40 mM DTT, 2 mM spermidine, 100 mg/mL BSA, 7 mM each NTP (NEB), 2 U/µL RNAsin, and 5 U/µL T7 polymerase (NEB). The transcription was performed for 3 h at 37 °C. DNA template was removed from the transcription mix by treatment with 0.08 U/µL DNAse I in the presence of the corresponding buffer at 37 °C for 30 min. The reaction was stopped by EDTA addition up to 20 mM and heating at 75 °C for 10 min. The transcribed fragment was called AMV RNA3Δ (the sequence is presented in SM, Section S1), and it was purified by the RNA cleanup kit according to the manufacturer’s manual. The integrity of the RNA was assessed by electrophoresis in 1% agarose gel in Tris/Borate/EDTA (TBE) buffer (SM, Section S2). The concentration of AMV RNA3 was measured by NanoDrop ND-2000 spectrophotometer (ThermoFischer Scientific, Waltham, MA, USA).

### 3.3. Preparation of Conjugate of Gold Nanoparticles with Antibodies

Conjugation of gold nanoparticles (GNPs) with mouse monoclonal antibodies specific to fluorescein (anti-FAM) was prepared as described in [9]. Briefly, to synthesize the GNP with a diameter of 22 nm, 1 mL of 1% HAuCl_4_ and 95 mL of deionized water were mixed and heated to a boiling point, then 4 mL of 1% sodium citrate was added and boiled for 25 min. The final concentration of the GNP was 1 nM, which corresponded to an optical density at 520 nm (OD_520_) equal to 1.0. For the conjugate synthesis, anti-FAM and GNPs were adjusted to pH 9.0, mixed with a final ratio of 10 µg of anti-FAM per 1 mL of 1 nM GNP solution, incubated for 1 h at 20 °C, blocked with BSA to 0.25%, and separated from the unbinding proteins via centrifugation at 10,000× *g* for 30 min at 4 °C.

### 3.4. Preparation of Lateral Flow Test Strips

The GNP–anti-FAM conjugate (OD_520_ = 4.0) was dispensed at 3.2 μL per 1 mm of strip width on a PT R5 fiberglass membrane. Streptavidin 1 mg/mL and goat anti-mouse IgG were dispensed at 0.15 μL per 1 mm of strip width on test and control zones of nitrocellulose membrane CNPC12 using an IsoFlow Dispenser (Imagene Technology, Lebanon, NH, USA). Test strips were assembled using the sample membrane GFB-R4, final adsorbing membrane AP045, and the abovementioned fiberglass and nitrocellulose membranes. The multi-membrane composites were cut and packed according to Byzova et al. [44].

### 3.5. Sample Collection and Characterization

Healthy (*n* = 3) potato plants and plants (potato, tobacco) artificially infected by AMV (*n* = 8) were grown in vitro and provided by Dr. Y.A. Varitsev and Dr. P.A. Galushko (A.G. Lorch Russian Potato Research Center, Kraskovo, Russia). For precise extraction of total nucleic acid, samples of potato leaves (150 mg) were homogenized by mortar and pestle. The extraction of total RNA from the samples was performed using a commercial total plant DNA/RNA extraction kit (Syntol, Moscow, Russia) according to the manufacturer’s protocols.

Crude extraction was performed by grinding 150 mg of plant material in a plastic bag with a ziplock containing plastic mesh. The extraction was performed in 1 mL of DNA/RNA-friendly 20 mM Tris-HCl pH 8.0, EDTA 0.2 mM (TE buffer) [45]. Potato virus X (PVX), potato virus Y (PVY), potato leafroll virus (PLRV), and potato virus S (PVS) were collected and purified by Dr. Y.A. Varitsev. Genomic RNA of these viruses was extracted by Syntol extraction kit. 

### 3.6. Primers and Probe Designs

Primers and probes for RPA were designed according to the recommendations of TwisDx (UK) [12]. Predictions of dimerizations were obtained using OligoCalc [46] and ThermoFisher’s Multiple Primer Analyzer (ThermoFisher Scientific) online software. 

### 3.7. Real-Time Quantitative PCR (qPCR) of AMV RNA3 Δ

The designed primers were checked in RT-qPCR, performed by Light Cycler 96 (Roche, Basel, Switzerland). Commercial premix (Evrogen) containing SYBR Green I, polymerase, dNTPs, and buffer was supplemented with 6 U/µL MMLV, 0.2 U/µL RNAse inhibitor, and 2 mM DTT. Primers were added in different combinations at a concentration of 200 nM. Transcribed AMV RNA3 Δ or total extract from plants was added in different concentrations. The PCR analysis comprised the RT stage at 42 °C for 20 min, then denaturation at 95 °C for 5 min, followed by 45 cycles. Each cycle contained denaturation at 95 °C for 30 s, primer annealing at 55 °C for 30 s, and elongation at 72 °C for 40 s. SYBR Green fluorescence was detected within the elongation stages. A cycle threshold (Ct) was computed automatically by Light Cycler software (Roche, Basel, Switzerland).

### 3.8. Two Variants of RPA-LFT for Detection of AMV RNA3 Δ

RPA reaction was performed as recommended by the manufacturer, with minor modifications. In the case of RPA based on labeled primers, 300 nM FAM- and biotin-labeled primers were used. In the case of a probe generating a labeled amplicon, 200 nM FAM-labeled probe, 300 nM unlabeled forward, and biotin-labeled primers were added in the RPA reaction. Primers of interest were added to the TwistDx rehydration buffer, then 6 U/µL MMLV reverse transcriptase and 0.2 U/µL RNAse inhibitor 10 mM DTT were appended. Then, 10 µL of a solution containing AMV RNA3, plant extracts, or control buffer was added to the mixture. In addition, TE buffer was used as a negative control and for the dilution of AMV RNA3. A lyophilized pellet from the nfoRPA kit was dissolved in the mix. To start the reaction, 14 mM magnesium acetate was added. RPA was carried out at 39 °C for 20 min using BioRad T100 Thermal Cycler (Hercules, CA, USA). After the reaction, 5 µL of the RPA mix solution was added to 65 µL of 50 mM phosphate-buffered saline, pH 7.4, 100 mM NaCl (PBS), and used as a sample for LFT. The test strip was submerged in the tested sample for 5 min at 20 °C or 37 °C. The qualitative results were estimated visually after 10 min.

To quantify the results, the test strips were scanned using a Canon 9000 F Mark II scanner (Canon, Tokyo, Japan), and the digital images were analyzed with a TotalLab TL120 (Nonlinear Dynamics, Newcastle upon Tyne, UK). The dependences of the color intensity of the LFT test line on the AMV RNA3 concentration were constructed using Origin Pro 9.0 (OriginLab, Northampton, MA, USA). The measured value of 2 arbitrary units according to the appearance of the visible test zone. For the calibration curve, each point was measured in duplicate at least.

## 4. Conclusions

We compared two approaches to obtain labeling RPA products for LFT detection— using only primers and primers with the oligonucleotide probe. The probe-based approach demonstrated no false-positive signal but had lower sensitivity than the approach with labeled primers. We found that the presence of the DNA/RNA of the potato plant in the RT-RPA-LFT assay eliminated nonspecific background signal and improved the specificity and sensitivity of the approach based on labeled primers. As a result, a primer-based RT-RPA-LFT assay that detected 10^3^ copies of AMV RNA3 was developed. The total time of amplification and detection was 30 min. Using the primer-based approach, the first test that clearly identifies AMV-infected plants in resource-limited conditions was developed. The test can be applied in field experiments because it complies with the basic requirements for an RPA kit—labeled primers, room temperature for LFT, and rapid and equipment-free extraction of plant total DNA/RNA. The obtained results can be the main reason for choosing simple labeling with primers for RT-RPA-LFT for the detection of other pathogens.

## Figures and Tables

**Table 1:** Sequences of primers used in the research.

Name	5′-3′ Sequence	5′ Modification
AMV F1 *	CCATCATGAGTTCTTCACAAAAG	FAM/none
AMV R1 *	TCGTCACGTCATCAGTGAGAC	biotin
AMV F3	ATTACTTCCATCATGAGTTCTTCACAAAAG	FAM/none
AMV R3	CATCCTCAGTCGTCACGTCATCAGTGAGAC	biotin
AMV F4	TTACGCAAAGCTCAACTGCCGAAGCCTCC	FAM/none
AMV R4	GAATCTCACGCCGAGCCCATTAAAAGAG	biotin
AMV THF probe	(FAM)- AAACCGACGAATACTATACTGCCACAGACG-(THF)- GCTGCGTGTGGCAA-Pi	FAM

* Primers were proposed by Xu and Nie [34].

## Data Availability

The data presented in this study are available on request from the corresponding author.

## Supplementary Materials

The following are available online at https://www.mdpi.com/article/10.3390/ijms222111885/s1.
