# Supplementary material for: Recombinase Polymerase Amplification Assay with and without Nuclease-Dependent-Labeled Oligonucleotide Probe
Source: Int J Mol Sci. 2021 Nov 2;22(21):11885. doi: 10.3390/ijms222111885 (PMC8584857; doi:10.3390/ijms222111885)
Supplement: Supplementary file 1 [file ijms-22-11885-s001.zip › ijms-1431609-supplementary.pdf]

SUPPLEMENTARY MATERIALS

Recombinase polymerase amplification assay with and without nuclease-dependent-labeled oligonucleotide probe

Aleksandr V Ivanov, Irina V Safenkova, Anatoly V Zherdev, Boris B Dzantiev*

A.N. Bach Institute of Biochemistry, Research Centre of Biotechnology of the Russian Academy of Sciences, 119071 Moscow, Russia

*Corresponding author, e-mail address: dzantiev@inbi.ras.ru

CONTENT

Section 1. Primers' sequences for target AMV gene and the amplicons obtained with them 2

Section 2. In vitro transcription of target AMV RNA3Δ 3

Section 3. Primers' selection by RT-qPCR 4

Section 4. Results of RT-RPA-LFT and their sigmoidal fits 6

Section 5. Verification of healthy and infected samples by RT-qPCR 7

References 8

Section 1. Primers' sequences for target AMV gene and the amplicons obtained with them

Table S1. Primers for AMV detection designed in previous researches.

Name	Sequence 5'-3'	Length, nt	Target RNA of AMV genome	Predicted dimers	Amplicon length, bp	Ref
AMV CP1 F	TCCATCATGAGTTCTTCAC	19	RNA 3	No	752	[1]
AMV CP1 R	AGGACTTCATACCTTGACC	19				
AMV F1	CCATCATGAGTTCTTCACAAAAG	23	RNA 3	No	351	[2]
AMV R1	TCGTCACGTCATCAGTGAGAC	21				
AMV F2	ATCATGAGTTCTTCACAAAAGAA	23	RNA 3	No	669	[2]
AMV R2	TCAATGACGATCAAGATCGTC	21		Yes		
AMV RNA2 F2	CGTTCCTACCATAAGGGATGTC	22	RNA 2	No	795	[3]
AMV RNA2 R2	CTTTTGTCAAACTTCGAAAAGTC	23		Yes		
AMV CP 214F	GCGAGATTCCTCTACAGTTT	20	RNA 3	No	363	[4]
AMV CP 576R	GACCCAAACTTCGTTGAATC	20		Yes		

Sites of designed primers recognition:

>AMV RNA3 Δ gene

CA**ATTACTT**CCATCATGAGTTCTTCACAAAAGAAAGCTGGTGGGAAAGCTGGTAAACCTACTAAACGTT
CTCAGAACTATGCTGCCTTACGCAAAGCTCAACTGCCGAAGCCTCCGGCGTTGAAAGTCCCGGTTGTAA
AACCGACGAATACTATACTGCCACAGACGGGCTGCGTGTGGCAAAGCCTCGGGACCCCTCTGAGTCTGA
GCTCTTTTAATGGGCTCGGCGTGAGATTCCTCTACAGTTTTCTGAAGGATTTCGCGGGACCTCGGATCCT
CGAAGAGGATCTGATTTACAGGATGGTGTTTTCCATAACACCGTCCTATGCCGGCACCTTTTGTCTCACT
GATGACGTGACGACTGAGGATGGTAGGGCCGTTGCGCATGGTAATCCCATGCAAGAATTTCCTCATGGC
GCGTTTCACGCTAATGAGAAGTTCGGGTTTGAGTTGGTCTTCACAGCTCCTACCCATGCGGGAATGCAA
AACCAAAATTTCAAGCATTCCTATGCCGTAGCCCTCTGTCTGGACTTCGACGCGCAGCCTGAGGGATCTA
AAAATCCCTCATACCGATTCAACGAAGTTTGGGTCGAGAGAAAGGCGTTCCCGCGAGCAGGGCCCCTCC
GCAGTTTGATTACTGTGGGGCTGCTCGACGAAGCTGACGATCTTGATCGTCATTGATGTACCCCATTAAT
TTGGGATGCCAAAGTCATTTGATGCTGACCTCCACTGGGTGGATTAAGGTCAAGGTATGAAGTCCTATT
CGCTCCTGATAGGATCGACTTCATATTGCTTATATATGTGCTAACGCACATATATAAATGCTCATGCAAAA
CTGCATGAATGCCCCTAAGGGATGC

Primer annealing legend: F1 , **F3,** F4, THF FAM, THF block, R4, R1, R3

Table S2. Lengths of potential amplicons, bp.

	F1	F3	F4	THF FAM
R1	351	358	274	223
R3	360	367	283	232
R4	227	243	150	99

Section 2. In vitro transcription of target AMV RNA3Δ

In-vitro transcription of AMV RNA3Δ was performed as described in the Material and Methods Section. A DNA template with a length of 900 bp was synthetized by PCR. 2.5 µL (1/20 volume) of transcription mix taken after 3h of the reaction was analyzed by electrophoresis in 2% agarose-TBE gel (**Figure S1A**). Target RNA (859 nt) was detected as band and corresponded 400-500 bp of DNA ladder. The mix was treated by DNAseI and then purified though the RNA cleanup kit. 50 ng RNA was analyzed by electrophoresis in 2% agarose-TBE gel (**Figure S1B**).

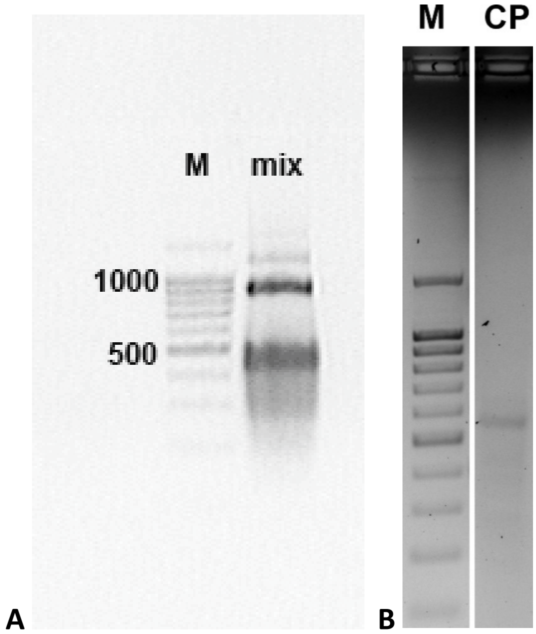

Figure S1. Electrophoresis of RNA corresponding CP gene of AMV (AMV RNA3Δ) in 2% agarose gel in TBE. A. Transcription mix after 3h of the reaction: M – DNA ladder marker (Evrogen), mix – transcription mix. B. Purified AMV RNA3Δ after DNAseI treatment and cleaning by RNA kit (NEB). M - DNA ladder marker (Evrogen), CP – RNA of coat protein or AMV RNA3Δ.

Section 3. Primers' selection by RT-qPCR

The transcribed AMV RNA3 was sequentially diluted from 10^7 to 10 copies and used as a template for sensitivity assessment. All the tested pairs demonstrate a saturation signal at a low concentration of AMV RNA3 or mQ background in RT-qPCR. It should cause the formation of by-products or cross-reaction between the primers. All tested pairs showed non-pronounced linear concentration dependence (**Figure S2**) that impaired the quantification of the AMV RNA3 target. Feasibly, the low sensitivity of RT-qPCR (10^4–10^5 copies for different pairs) could be caused by the twisting of the RNA.

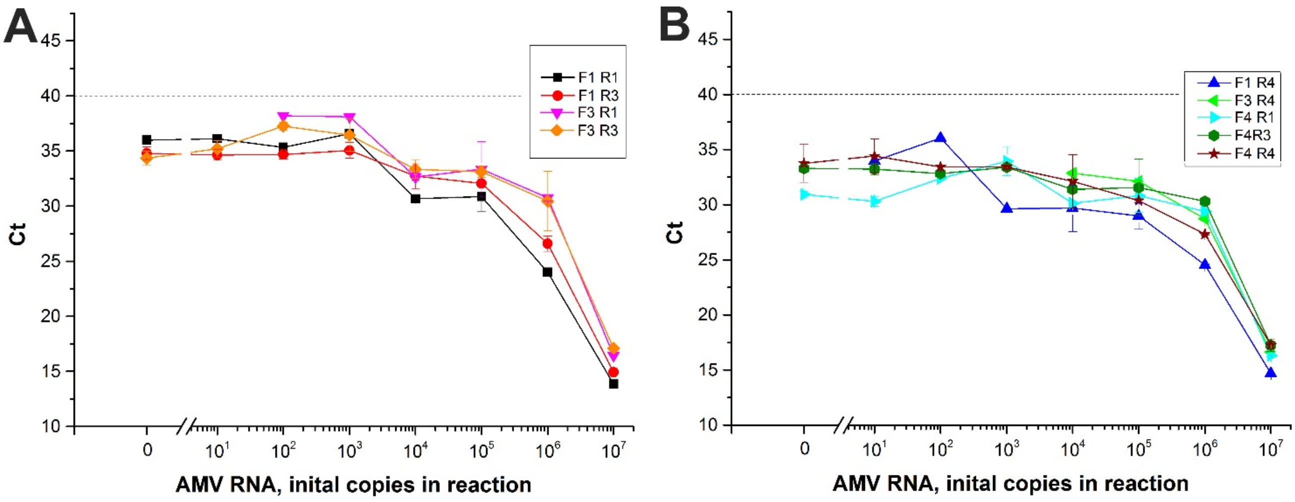

Figure S2. Results of RT-qPCR of target AMV RNA3Δ obtained with different combinations of primers. A. RT-qPCR curves obtained with F1-R1, F1-R3, F3-R1, and F3-R3 primers' combination. B. RT-qPCR curves obtained with F1-R4, F3-R4, F4-R1, F4-R3, and F4-R4 primers' combination.

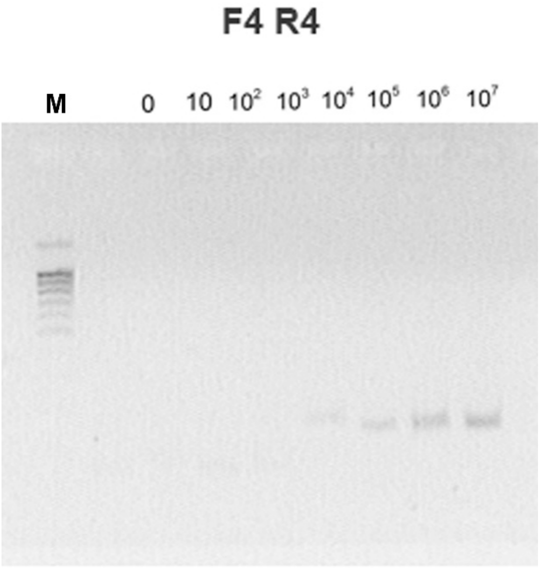

Figure S3. Electrophoresis of samples after RT-qPCR with the F4-R4 pair in presence of different amounts of the target AMV RNA. M – DNA ladder marker (Evrogen).

Section 4. Results of RT-RPA-LFT and their sigmoidal fits

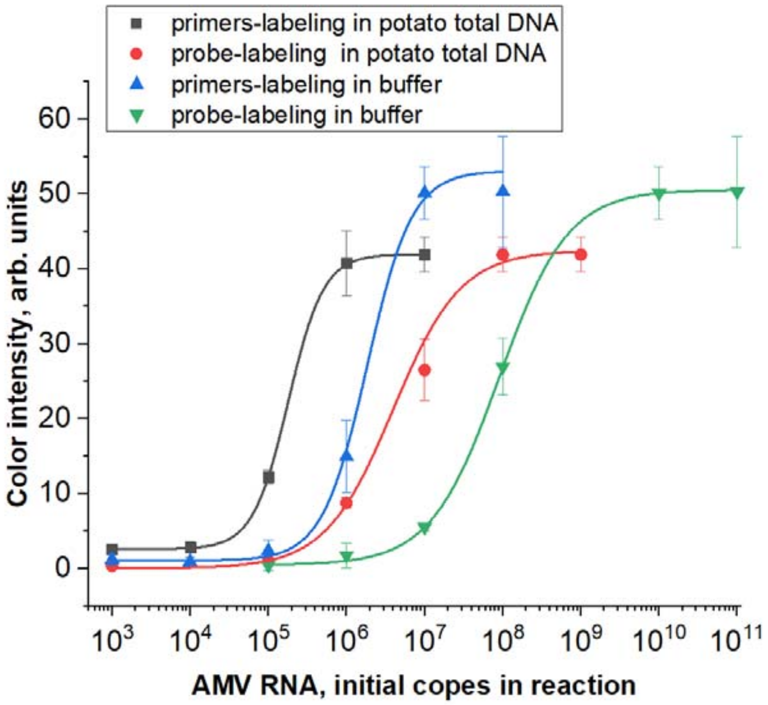

Figure S4. Results of RT-RPA-LFT for different amount of synthetic AMV RNA3 performed by primers-labeling and probe-labeling methods. The target RNA diluted in TE buffer or in potato total DNA. Curves were obtained by sigmoidal fitting.

Table S3. Parameters of sigmoidal fits for results of RT-RPA-LFT for different amount of synthetic AMV RNA3 performed by primers-labeling and probe-labeling methods.

Model	Logistic			
Equation	y = A2 + (A1-A2)/(1 + (x/x0)^p)			
Plot	**primers-labeling in potato total DNA**	**probe-labeling in potato total DNA**	**primers-labeling in buffer**	**probe-labeling in buffer**
A1	2.6 ± 0.03	0.04 ± 0.41	1.06 ± 0.24	0.47 ± 0.21
A2	41.97 ± 0.23	42.47 ± 1.73	53.1 ± 3.48	50.57 ± 1.11
x0 (**half-maximum binding**)	1.8E5 ± 0.8E5	4.0E6 ± 0.9E6	1.8E6 ± 0.5E6	9.0E7 ± 0.9E7
p	1.92 ± 0.14	1.0 ± 0.15	1.52 ± 0.38	0.99 ± 0.05
R-Square	0.99998	0.99785	0.99769	0.99923

Section 5. Verification of healthy and infected samples by RT-qPCR

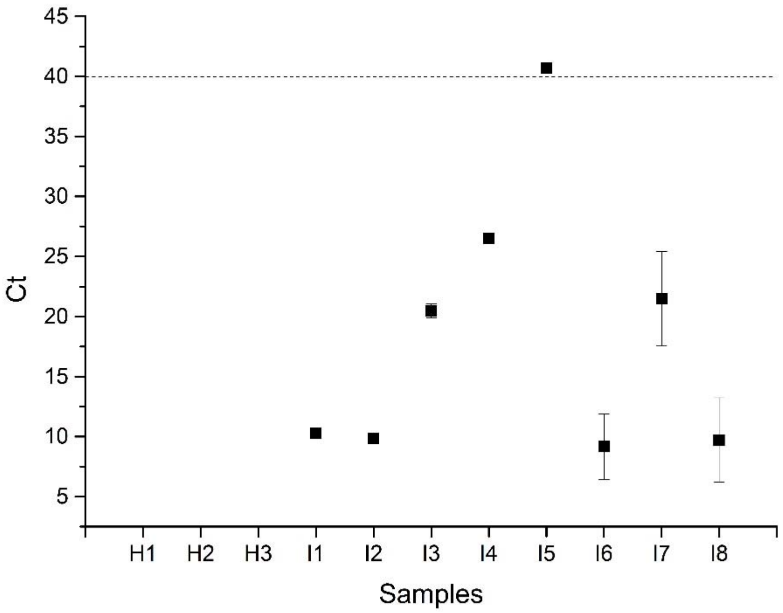

Figure S5. Ct values of samples extracted from healthy (H) and infected (I) plants. The dashed line represents detection limit.

References

1. Finetti Sialer, M.; Di Franco, A.; Papanice, M.A.; Gallitelli, D. Tomato necrotic yellows induced by a novel strain of alfalfa mosaic virus. *Journal of Plant Pathology* **1997,** 79, (2), 115-120.
2. Xu, H.; Nie, J. Identification, Characterization, and molecular detection of Alfalfa mosaic virus in potato. *Phytopathology* **2006,** 96, (11), 1237-42.
3. Abdel Aleem, E. E.; Taha, R. M.; Fattouh, F. A., Biodiversity and full genome sequence of potato viruses Alfalfa mosaic virus and potato leaf roll virus in Egypt. Journal of biosciences (*Z Naturforsch C)* **2018,** 73, (11-12), 423-438.
4. Samarfard, S.; Bejerman, N.E.; Sharman, M.; Trucco, V.; Giolitti, F.; Dietzgen, R.G. Development and validation of PCR assays for detection of alfalfa dwarf disease-associated viruses in Australian lucerne pastures. *Australasian Plant Pathology* **2017,** 47, 215-225.
